# Clinical / Therapeutic Name: Care and treatment of prisoners with intellectual disabilities

**DOI:** 10.1192/j.eurpsy.2024.69

**Published:** 2024-08-27

**Authors:** V. Tort Herrando, Forensic Psychiatric Section

**Affiliations:** ^1^Penitentiary Psychaitry, Parc Saniatria Sant Joan de Deu, Sant Boi de Llobregat (Barcelona), Spain

## Abstract

**Abstract:**

Care and treatment of prisoners with intellectual disabilities I will describe what is the care, from different points of view when an offender with intelledtual disabilities entry in the penitentiary system. As a vulnerable population, people with intellectual disabilities have to be treated in a more specific manner, and both prison managers and clinciacal staff have to be aware of of that. This prisoners, sometimes, also belongs to another vulnerable population ( illegal inmigration, fenmales, ehtnic groups ,etc) that make this cases as a complex ones.  The care have to be as a comprehensive, with thr higest standards of care a nd avoid neglicence en treating this cases. We have to emphasize about rehablitation and a goodl coordnation with the intellectual disability community services to avoid relapse and recidivism

**Disclosure of Interest:**

None Declared

